# Transcriptional profiling identifies differential expression of long non-coding RNAs in Jo-1 associated and inclusion body myositis

**DOI:** 10.1038/s41598-017-08603-9

**Published:** 2017-08-14

**Authors:** Philip D. Hamann, Benoit T. Roux, James A. Heward, Seth Love, Neil J. McHugh, Simon W. Jones, Mark A. Lindsay

**Affiliations:** 1 Department of Pharmacy and Pharmacology, University of Bath, Claverton Down, Bath, BA2 7AY UK; 2 Royal National Hospital for Rheumatic Diseases, Upper Borough Walls, Bath, BA1 1RL UK; 3 Barts Cancer Institute, Queen Mary University of London, London, EC1M 6BQ UK; 4 MRC ARK Centre for Musculoskeletal Aging Research, University of Birmingham, Birmingham, B15 2TT UK; 5 Dementia Research Group, Institute of Clinical Neurosciences, School of Clinical Sciences, University of Bristol, Bristol, BS16 1LE UK

## Abstract

Myositis is characterised by muscle inflammation and weakness. Although generally thought to be driven by a systemic autoimmune response, increasing evidence suggests that intrinsic changes in the muscle might also contribute to the pathogenesis. Long non-coding RNAs (lncRNAs) are a family of novel genes that regulate gene transcription and translation. To determine the potential role of lncRNAs, we employed next generation sequencing to examine the transcriptome in muscle biopsies obtained from two histologically distinct patient populations, inclusion body myositis (IBM) and anti-Jo-1-associated myositis (Jo-1). 1287 mRNAs and 1068 mRNAs were differentially expressed in the muscle from Jo-1 and IBM patients, respectively. Pathway analysis showed the top canonical pathway in both Jo-1 and IBM was oxidative phosphorylation and mitochondrial dysfunction. We identified 731 known and 325 novel lncRNAs in the muscles biopsies. Comparison with controls showed 55 and 46 lncRNAs were differentially expressed in IBM and Jo-1 myositis, respectively. Of these, 16 lncRNAs were differentially expressed in both IBM and Jo-1 myositis and included upregulated *H19*, *lncMyoD* and *MALAT1*. Given that these are known to regulate muscle proliferation and differentiation, we speculate that changes in lncRNAs might contribute to the phenotypic changes in Jo-1 and IBM myositis.

## Introduction

Idiopathic inflammatory myopathies (IIM) represent a group of autoimmune inflammatory muscle conditions whose clinical symptoms include muscle weakness and inflammation (myositis), often accompanied by characteristic cutaneous manifestations and interstitial lung disease^1^. IIM can be broadly divided into polymyositis (PM), dermatomyositis (DM) and inclusion body myositis (IBM) based on clinical symptoms and histopathological features. PM and DM are also commonly sub-classified on the basis of the presence of specific autoantibodies. Those against the cytoplasmic aminoacyl-tRNA synthetases, including Jo-1 (anti-histidyl-tRNA synthetase), are the most frequently detected in adult patients, and the associated disease is described as anti-synthetase syndrome (ASS)^2^.

The mechanisms underlying the development of these inflammatory myopathies remain unknown, although T-cell infiltration and the presence of autoantigens and MHC class I (and occasionally MHC class II) antigens in the muscle, highlights the potential importance of the adaptive immune response and autoimmunity. These histological observations have guided the treatment strategies that generally involve the use of anti-inflammatory and immunosuppressive drugs such as glucocorticoids, azathioprine and methotrexate. However, a parallel non-immune aspect to this disease is implied by the observation that many patients, particularly those with IBM, do not response to glucocorticoids, that the degree of muscle inflammation does not correlate with muscle weakness and that disease symptoms return upon drug removal^3^. Furthermore, a recent study in a mouse model of inflammatory myopathy has demonstrated that the onset of muscle weakness and metabolic disturbances occurred prior to the appearance of infiltrating mononuclear cells^4^. These studies suggest that intrinsic changes in the skeletal muscle may drive these idiopathic myopathies.

The advent of next generation sequencing has revolutionised our understanding of the transcriptome by allowing the quantitation of protein-coding mRNAs and non-coding RNA expression. This approach has identified many novel families of non-coding RNAs that are known to impact upon both physiological and pathological responses through regulating the transcription, translation and turnover of mRNA^5, 6^. These non-coding RNAs can be broadly divided into three groups: house-keeping, short and long non-coding RNA. House-keeping non-coding RNAs typically have short sequences, conserved secondary structures and may be transcribed by RNA polymerase I, II or III. These include well characterised families such as the transfer RNAs and ribosomal RNAs that are involved in translation. The small non-coding RNAs have a length <200 nucleotides, with the three best studied families being the piwi-associated RNAs, endogenous small interfering RNAs and microRNAs, which all employ argonaute proteins to guide sequence-specific regulation of transcription and/or translation. However, the majority of non-protein coding RNA are defined as long non-coding RNAs (length >200 nucleotides) with >30,000 currently annotated in the GenCode database^7^. These are traditionally classified according to their evolutionary origin and/or their position relative to protein-coding mRNAs and include antisense, pseudogenes and long intergenic non-coding RNAs (lincRNAs). Functional studies indicate that lncRNAs have widespread actions upon mRNA expression through regulating protein-protein and protein-DNA interactions^8, 9^.

Although there is extensive evidence showing that miRNAs are central regulators of skeletal muscle development, little is known about the function of lncRNAs and whether changes in their expression are linked to diseases such as myositis^10, 11^. Interestingly, recent studies in mice have identified a number of lncRNAs that regulate skeletal muscle proliferation and differentiation. These include *H19* that is located within an imprinted region and paternally expressed during fetal development. In adults, *H19* expression is repressed in all tissues except skeletal muscle, where it has been shown to regulate differentiation, glucose metabolism and the expression of other imprinted genes including insulin-like growth factor (*Igf2*)^12–16^. A number of publications have also independently identified a lncRNA that is located upstream of the MyoD transcription factor and named either ^*DRR*^ *RNA* ^17^, upstream non-coding (*MUNC*)^18^ or *LncMyoD* ^19^, which is also known to regulate muscle proliferation and differentiation^18, 19^. A syntenic version of *LncMyoD/MUNC* has been identified in humans and called *hLncMyoD*.

Although previous studies have attempted to understand the aetiology of myositis by examining the profile of mRNA^20^ and miRNA expression^21^, none have investigated the changes in lncRNAs. This is particularly relevant given the emerging evidence that lncRNAs regulate a range of skeletal muscle phenotypes that could be linked to myositis including proliferation and differentiation. To address this question, we have undertaken next generation sequencing to examine the profile of lncRNA expression in control muscle biopsies and those obtained from individuals with IBM and Jo-1 myositis. These studies have identified a range of lncRNAs that are differentially expressed in myositis including *H19*, *lncMyoD*, *NEAT1*, *PVT1*, *MEG3* and *MALAT1*. Overall, our data suggest that changes in the expression of lncRNAs may contribute to the phenotype of myositis.

## Results

### Clinical Characteristics

**Figure 1:**
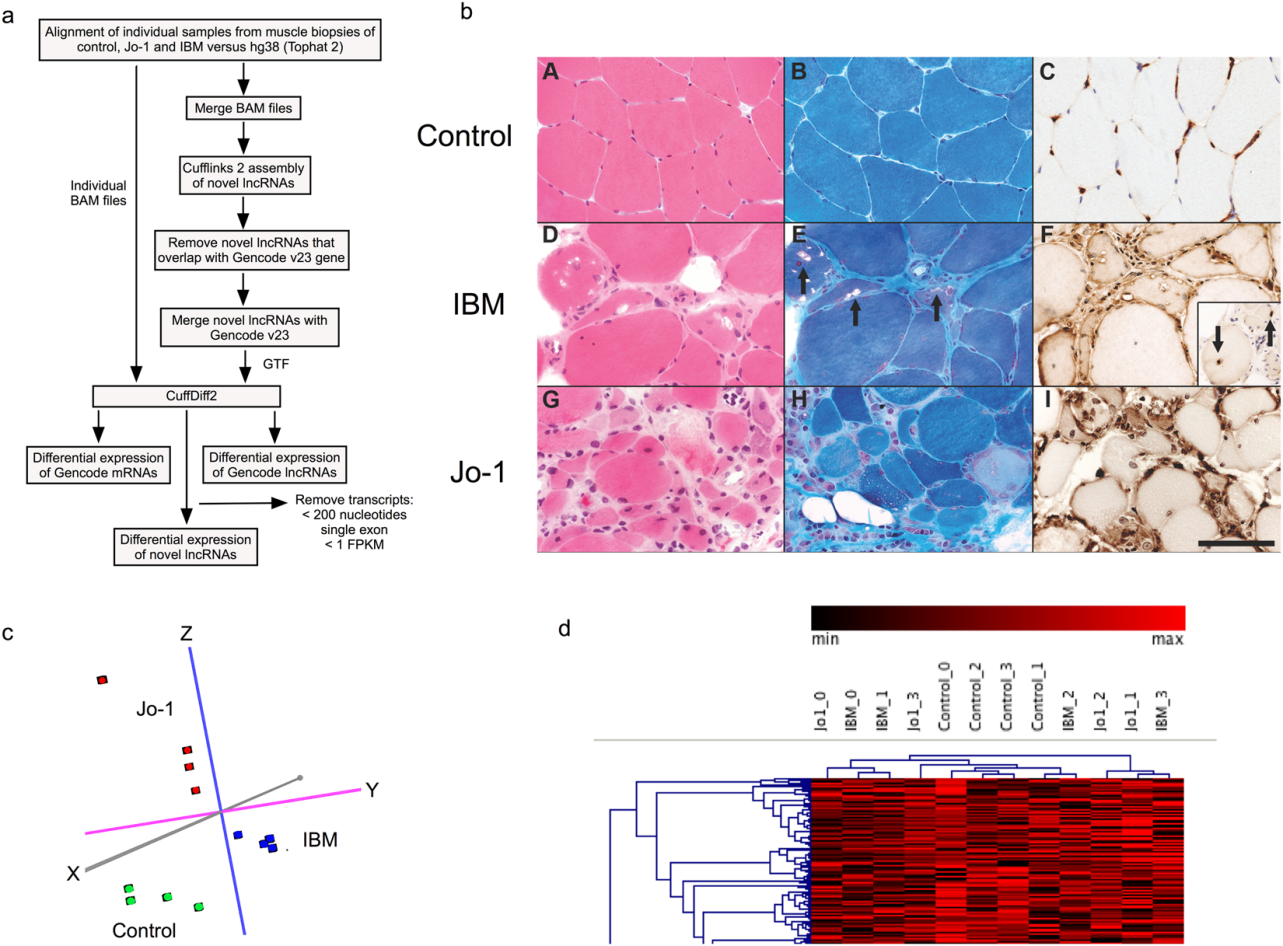
Muscle biopsy analysis. (**a**) Description of bioinformatics analysis pathway, (**b**) Muscle histology where A-C shows normal muscle tissue from one of the controls. D-F shows classical features of IBM in one of the biopsies. G–I are sections from a muscle biopsy from one of the patients with Jo-1 myositis. A,D and G were stained with HE and B,E and H with modified Gomori trichrome. C,F and I were immunolabelled for β_2_-microglobulin. Inset in F shows inclusions immunolabelled for ubiquitin. The scale bar represents 50 μm, (**c**) Principle component analysis (PCA) of the mRNA data from control, IBM and Jo-1 myositis, (**d**) Non-supervised hierarchical cluster analysis of the mRNA data from control, IBM and Jo-1 myositis.

Myositis was diagnosed by clinical, histological, electron microscopic and immunohistochemical analysis of the biopsies (Fig. 1b and Table 1). IBM diagnosis was primarily based upon the detection of classical rimmed vacuoles and tubulofilamentous inclusions on electron microscopy, usually in association with occasional ragged red fibres. The diagnosis of anti-Jo-1 myositis was based on the presence of autoantibodies to anti-histidyl-tRNA synthetase (anti-Jo-1) and histological demonstration of inflammation and degeneration. Control specimens were all muscle biopsies demonstrating no inflammatory or degenerative features. The mean ages of the groups were 51 years (41–65), 61 years (50–66) and 50.6 years (range 41–64 years) for control, IBM and Jo-1 myositis patients respectively. The ratio of male to female patients was 1:4 for the control group, 0:5 in the IBM group and 1:4 in the anti-Jo-1 group.

**Table 1:** Histological description of individual patient biopsies.

Diagnosis	Clinical information	Biopsy Findings
Jo-1	Muscle pain and weakness	Occasional mildly or moderately atrophic fibres.
Jo-1	Muscle pain, weakness and shortness of breath	Atrophic fibres and endomysial inflammatory cells with surface expression of beta 2 microglobulin on muscle fibres and complement terminal attack complex.
Jo-1	Muscle pain and weakness	Scattered moth-eaten fibres, myofibrillar disarray with increased variation in fibre size and endomysial cell debris.
Jo-1	Proximal upper and lower limb muscle pain with elevated creatinine kinase	Necrotic and regenerating fibres with atrophic fibres, split fibres and fibres with central nuclei. Endomysial inflammatory infiltrate.
Jo-1	Progressive bilateral leg swelling with elevated creatinine kinase and inflammatory markers.	Necrotising inflammatory myositis with inflammatory cell infiltration and enlarged central vacuoles.
IBM	Trunk, upper and lower limb weakness.	Inflammation, degenerative and regenerating fibres, multiple rimmed vacuoles; with inclusions
IBM	Upper and lower limb weakness and wasting with difficulty swallowing.	Atrophic fibres and fibres containing rimmed vacuoles, and inflammatory cell infiltrate.
IBM	Upper limb muscle pain and tenderness and elevated creatinine kinase.	Inflammatory changes and infiltrate with fibre atrophy, degeneration, regeneration, adipose tissue replacement and myofibrillar disorganization. Increase in endomysial collagen.
IBM	Pain and weakness in proximal lower limb	Atrophic, necrotic and regenerating fibres with adipose tissue replacement. Focal fibrosis, endomysial inflammation, internal nuclei and lymphocytic. Rimmed vacuoles with up-regulation of beta 2 microglobulin.
IBM	Muscle pain and weakness	Hypertrophic and atrophic fibres embedded in fibrotic stroma. Necrotic fibres engulfed by macrophages and endomysial lymphocytic infiltration. Rimmed vacuoles and ragged red fibres and inclusions in myofibres.

To characterise these samples at the molecular level, we determined the profile of mRNA expression in individual biopsies by use of CuffNorm. Principle component analysis (PCA) supported the clinical and histological classification and showed that these biopsies could be clearly divided into control, IBM and Jo-1 myositis groups (Fig. 1c). Analysis by hierarchical clustering grouped the control samples but was unable to distinguish between IBM and Jo-1 samples (Fig. 1d).

### Differentially expression of protein-coding genes in Jo-1 and inclusion body myositis

**Figure 2:**
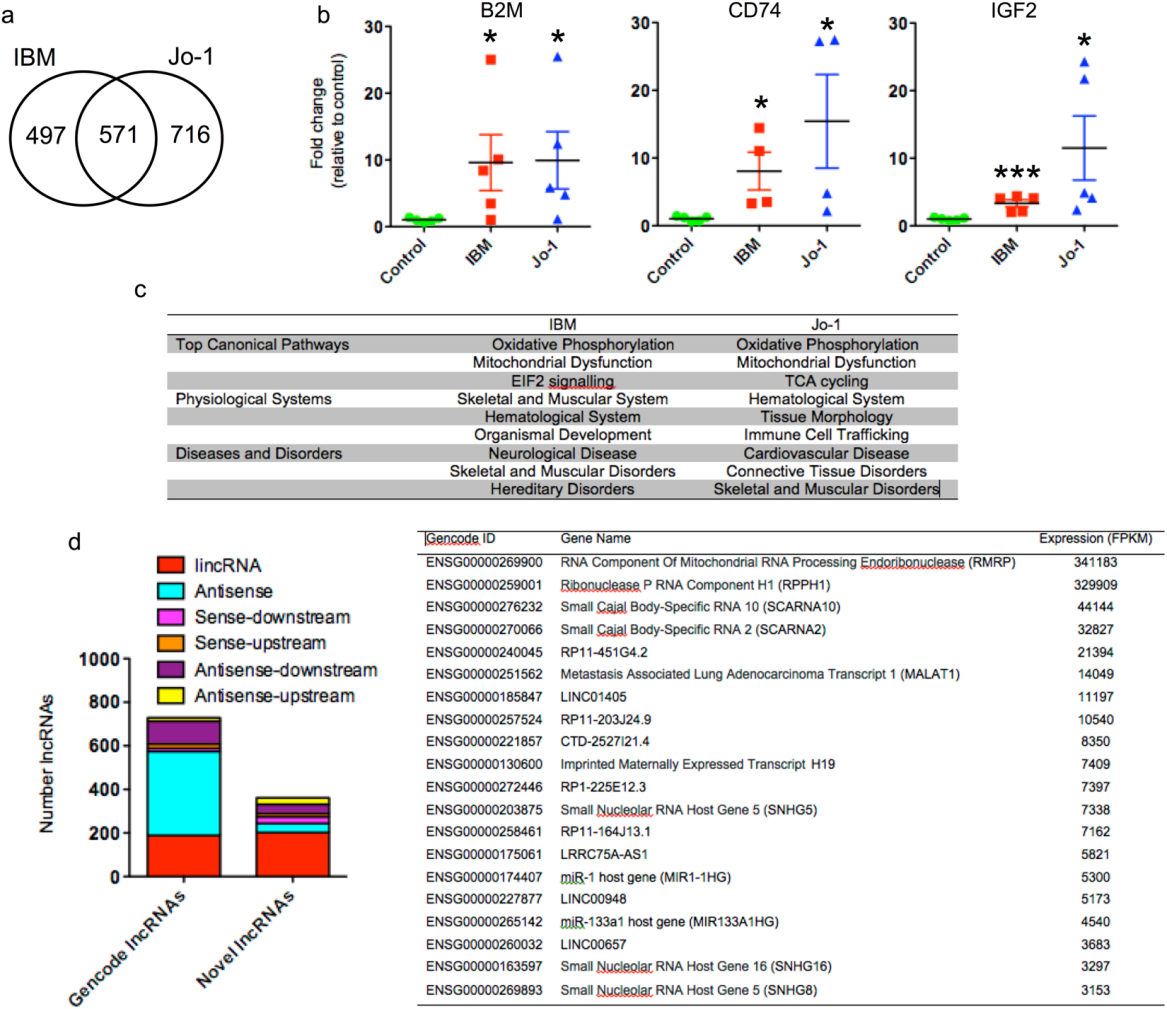
Analysis of mRNA and lncRNA expression data in control, IBM and Jo-1 myositis. (**a**) Venn diagram showing overlap between differentially expressed mRNAs in IBM and Jo-1 myositis, (**b**) Changes in levels of β_2_-microglobulin (B2M), CD74 and IGF2 mRNA expression were confirmed by qRT-PCR. The data are presented as the mean ± SEM of 4–5 patients where *p < 0.05 and ***p < 0.001 (Mann-Whitney U-test). (**c**) Pathway analysis of differentially expressed mRNAs in IBM and Jo-1 myositis, (**d**) Classification of Gencode and novel lncRNA in skeletal muscle and, (**e**) List of most highly expressed lncRNA in control skeletal muscle.

To characterize potential differences at the transcriptional level, we examined the profile of mRNA expression (FDR < 0.05) between control and IBM or Jo-1 samples, as well as any differences between IBM and Jo-1 (Fig. 2a and Supplemental Table 1). These studies showed differential expression of 1068 mRNAs (598 increased, 470 decreased) in IBM and 1287 (893 increased, 394 decreased) in Jo-1 myositis, compared with controls (Supplemental Table 1). Comparison of these lists revealed 571 that were differentially expressed in both Jo-1 and IBM (Fig. 2a) indicating that these samples have similar transcriptional landscapes, an observation supported by PCA analysis (Fig. 1c) and hierarchical clustering (Fig. 1d). By qRT-PCR, we were able to confirm the sequencing data by showing significant up-regulation in expression of CD74, β_2_-microglobulin (B2M) and insulin growth factor 2 (IGF2) in both Jo-1 and IBM (Fig. 2b).

To elucidate the potential role of these differentially expressed genes we undertook pathway analysis using Ingenuity (http://www.ingenuity.com). Interestingly, the top canonical pathways in both IBM and Jo-1 samples were oxidative phosphorylation and mitochondrial dysfunction, supporting previous reports that both types of myositis are associated with abnormal mitochondrial metabolism (Fig. 2c). From a physiological perspective, IBM was linked to skeletal, muscular and hematological systems whilst Jo-1 was also associated with immune cell trafficking, indicating that this type of myositis maybe more inflammatory in nature. Unsurprisingly, the changes in gene expression in both disorders were associated with skeletal and muscle disease, although the most prominent pathways were neurological in IBM and cardiovascular in Jo-1 myositis (Fig. 2c).

### Profile of lncRNAs expression in skeletal muscle

Before investigating potential differences in the muscle from patients with IBM and Jo1 myositis, we examined the profile of lncRNA expression in control skeletal muscle. Due to the difficulty in definitively assigning reads to pseudogenes during alignment, these were excluded from the analysis and we instead focused upon lincRNAs and antisense lncRNAs. As previously^22, 23^, we divided the lncRNAs (including those annotated in Gencode) into 6 groups based upon their relative position to protein coding genes: antisense (overlapping a protein-coding gene on the opposite strand), antisense-upstream (within 5 kb and located upstream/opposite strand from of a protein-coding genes), antisense-downstream (within 5 kb and located downstream/opposite strand from of a protein-coding genes), sense-upstream (within 5 kb and located upstream/same strand from of a protein-coding genes), sense-downstream (within 5 kb and located downstream/same strand from of a protein-coding genes) and lincRNAs (located >5 kb from a protein coding gene)^22^. Using a cut-off of FPKM > 1 to identify those expressed at physiologically relevant levels, we identified 731 lncRNAs in the Gencode v23 database. These could be divided into 189 lincRNAs, 385 antisense, 105 antisense-downstream, 16 antisense-upstream, 15 sense-downstream and 19 sense-upstream (Fig. 2d). Following transcript assembly we were able to identify an additional 325 novel lncRNAs that could be divided into 202 lincRNAs, 42 antisense, 30 antisense-downstream, 6 antisense-upstream, 30 sense-downstream and 15 sense-upstream (Fig. 2d). Examination of control muscle data showed that the most highly expressed lncRNAs included RNA component of mitochondrial RNA processing (*RMRP*), Ribonuclease P RNA Component H1 (*RPPH1*), the imprinted and maternally expressed transcript *H19*, metastasis associated lung adenocarcinoma transcript 1 (*MALAT1*) and the host genes for miR-1 and miR-133A1, two miRNAs that are known to be highly expressed and important in the regulation of skeletal muscle (Fig. 2d).

### Differential expression of long non-coding RNAs in inclusion body and Jo1 myositis

**Figure 3:**
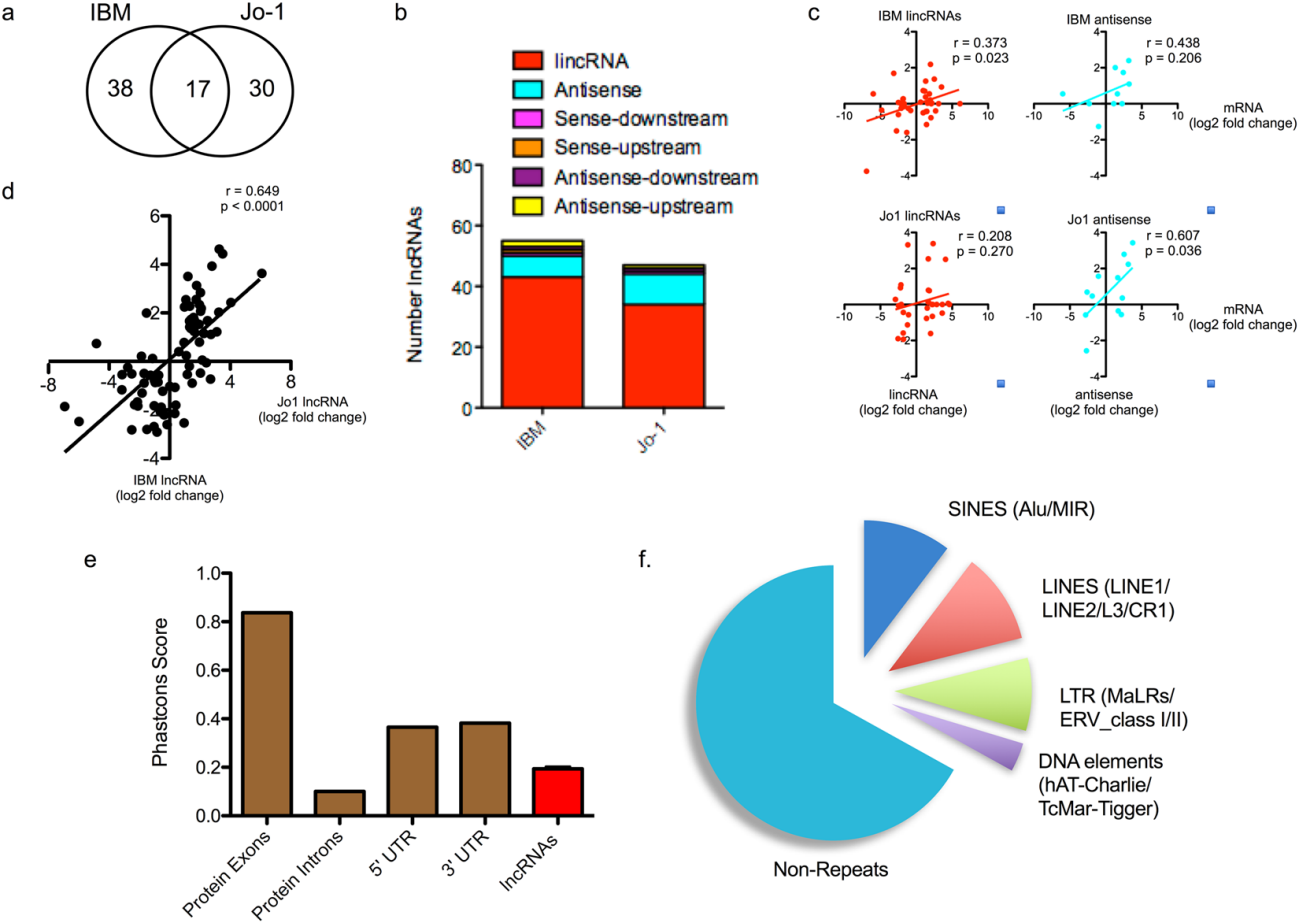
Characterisation of differentially expressed lncRNAs in control, IBM and Jo-1 myositis. (**a**) Venn diagram showing overlap between differentially expressed lncRNAs in IBM and Jo-1 myositis, (**b**) Classification of differentially expressed lncRNAs in IBM and Jo-1 myositis, (**c**) Correlation between differentially expressed lncRNAs and that of the nearest protein coding mRNA, (**d**) Correlation between differential lncRNA expression in IBM and Jo-1 myositis, (**e**) PhastCons analysis of the conservation of the differentially expressed lncRNA species in human and mouse cells compared with the exon, intronic and untranslated regions (UTRs) of protein coding genes and (**f**) Pie chart showing the % distribution of repeat sequences in the various sub-populations of lncRNAs across all 4 cell types with SINES = short interspersed nuclear elements, LINES = long interspersed nuclear elements and LTR = long terminal repeat.

There is now accumulating evidence that lncRNAs represent novel regulators of gene expression and that aberrant expression is associated with pathological responses. We therefore investigated whether changes in lncRNAs within the skeletal muscle were associated with IBM and Jo-1 myositis. We identified 55 lncRNAs (17 novel lncRNAs) and 48 lncRNAs (14 novel lncRNAs) that were differentially expressed in IBM and Jo1 patients, respectively (Supplemental Table 3). Overall, we identified 85 lncRNAs that were differentially expressed, with 38 and 30 lncRNAs selectively expressed in IBM and Jo-1 myositis alone and 17 of these in both (Fig. 3a).

In contrast to the lncRNA in control muscle, the majority of the differentially expressed lncRNAs were intergenic (i.e. lincRNAs (Fig. 3b)). Examination of the fold change in expression of lincRNA and antisense with their nearest protein coding gene showed some correlation, albeit weak, in the case of IBM lincRNAs (p = 0.023) and Jo1 antisense lncRNA (p = 0.036) (Fig. 3c). Pathway analysis of the nearest or overlapping (in the case of antisense) genes using DAVID (https://david.ncifcrf.gov) and of all genes within their 5 Mb flanking region (using GREAT at http://bejerano.stanford.edu/great/public/html/) found that these mRNAs were not associated with any specific pathways or ontological grouping. Interestingly, comparison of the fold change in lncRNA expression between IBM and Jo-1 myositis revealed robust correlation (p < 0.0001), with linear regression analysis showing comparable changes (slope = 0.55). This indicates that there are comparable changes in lncRNA expression in both IBM and Jo-1 myositis. Analysis of the evolutionary conservation of the combined lncRNAs showed that these were poorly conserved (Fig. 3e). Thus, using PhastCons (100-way vertebrate) which determines conservation on a 0–1 scale (1 being the most conserved), we obtained a value of 0.194 ± 0.009. This value was significantly greater than the 0.100 ± 0.003 for the intronic regions of protein coding genes (p < 0.0001 – Mann-Whitney U-Test) but considerably less than the value for exonic, 5′ and 3′ UTRs regions of protein coding genes at 0.837 ± 0.001, 0.365 ± 0.001 and 0.382 ± 0.001, respectively (Fig. 3e). These lncRNAs were also found to be enriched with repeat sequences (identified using repeatmasker.org) including 10.4% short interspersed nuclear elements (SINES), 10.6% long interspersed nuclear elements (LINES), 8.6% long terminal repeats (LTRs) and 3.8% DNA elements, leaving 66.6% of non-repeat sequence (Fig. 1f).

**Figure 4:**
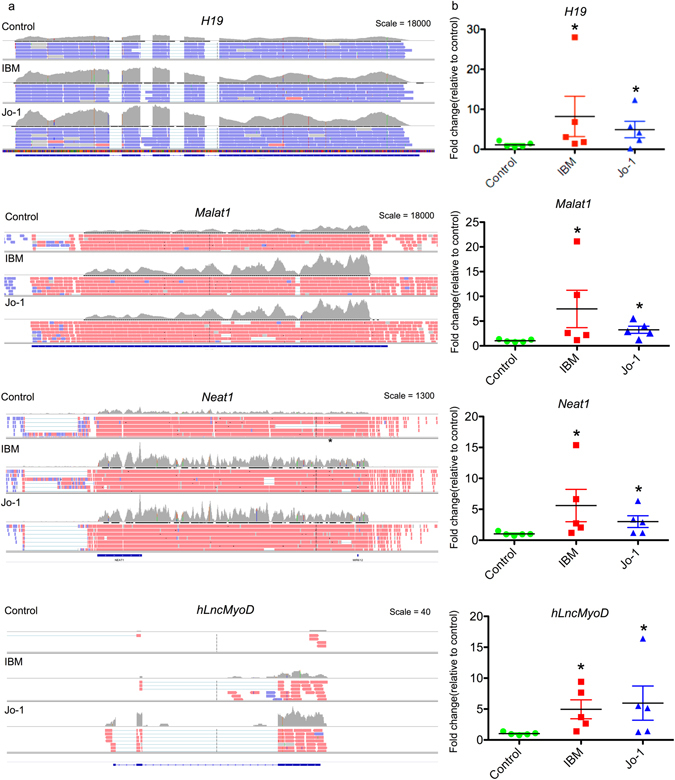
Differential expression of *H19*, *MALAT1*, *NEAT1* and *hLncMYoD* in control, IBM and Jo-1 myositis. (**a**) View from the Integrated Genome Viewer (IGV) of aligned sequence data for *H19*, *MALAT1*, *NEAT1* and *hLncMYoD*. Red and blue blocks represent reads aligned to the positive and negative strand of the DNA, respectively. The dark gray histogram represents the sum of the aligned sequencing reads along the genome, with the scale stated on the top right corner, (**b**) Changes in levels of *H19*, *MALAT1*, *NEAT1* and *hLncMYoD* were confirmed using qRT-PCR the data are the mean ± SEM of 5 patients where *p < 0.05 (Mann-Whitney U-test).

**Figure 5:**
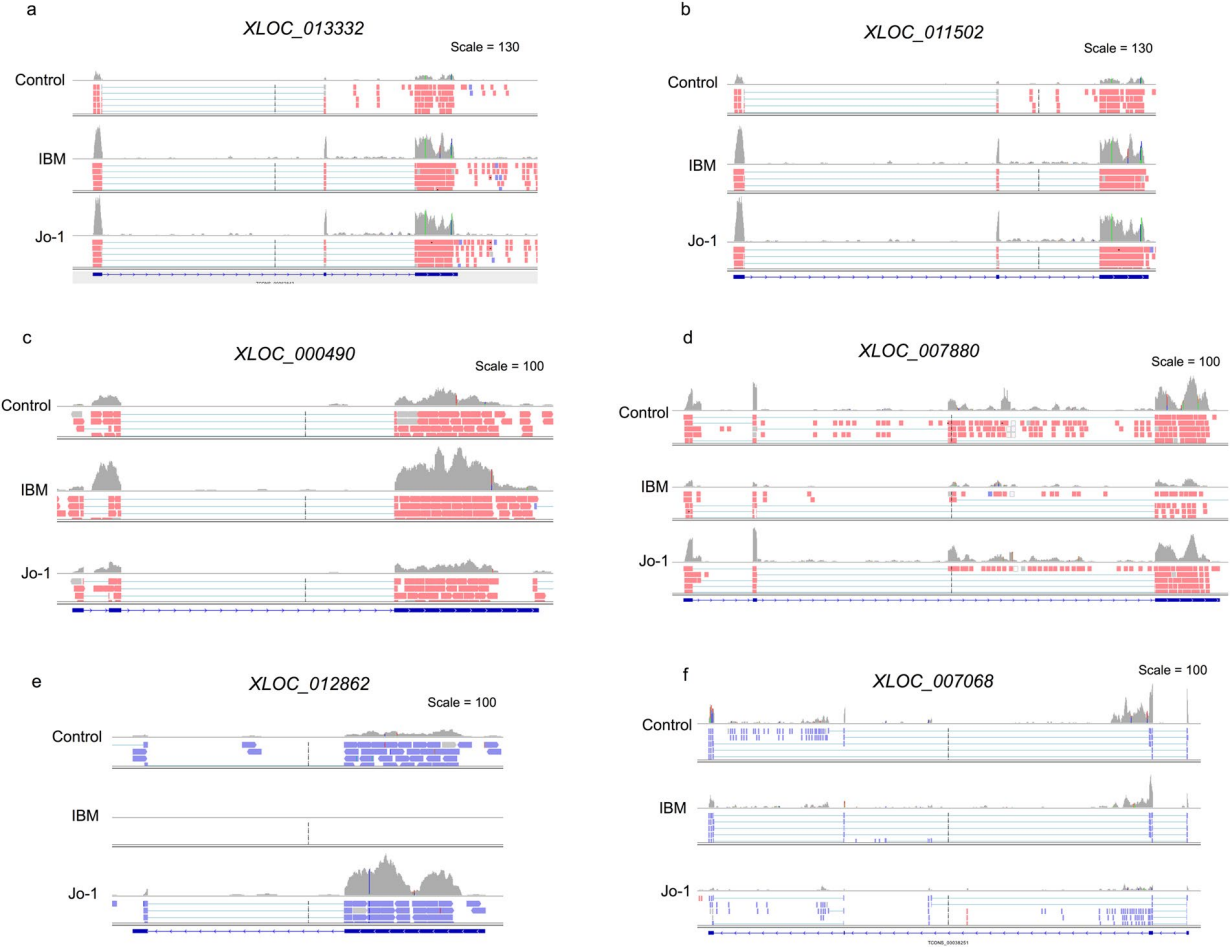
Differential expression of novel lncRNAs in control, IBM and Jo-1 myositis. View from the Integrated Genome Viewer (IGV) of aligned sequence data for novel lncRNA that are up-regulated (**a**,**c**,**e**) and down-regulated (**b**,**d**,**f**) in both IBM and Jo-1 myositis (**a**,**b**), IBM alone (**c**,**d**) and Jo-1 alone (**e**,**f**).

Of the 17 lncRNAs that were significantly changed in both IBM and Jo-1 myositis, the majority (13/17) were upregulated and included 4 novel lncRNAs. It is possible that these are markers or mediators of the phenotypic changes that are common to both diseases, such as muscle wasting. They included many well characterised lncRNAs such as *H19*, *lncMyoD*, noncoding nuclear-enriched abundant transcript 1 (*NEAT1*), plasmacytoma variant translocation 1 (*PVT1*), maternally expressed 3 (*MEG3*) and *MALAT1* (Supplemental Table 3 and Fig. 4a). Changes in the expression of these lncRNAs were confirmed by qRT-PCR (Fig. 4b). In contrast, it might be speculated that the 38 and 30 lncRNAs differentially expressed in IBM or Jo-1 myositis alone, could be drivers of the disease specific changes including the development of inclusion bodies in IBM or the inflammatory phenotype associated with Jo-1 myositis. Of the 38 lncRNAs in IBM, there was an equal distribution of up- and down-regulated lncRNAs (19 each) of which 13 were novel whilst for the 30 lncRNAs in Jo-1 myositis, 17 were up-regulated, 13 down-regulated and 10 were novel. As with the well characterised lncRNAs (Fig. 4), visual inspection of the novel lncRNAs that were both multi-exonic with well defined intron/exon boundaries, indicating that these might also have important functions (Fig. 5). Overall, these studies have identified a host of known and novel lncRNAs that are differentially expressed in IBM and Jo-1 myositis including *H19*, *lncMyoD*, *NEAT1*, *PVT1*, *MEG3* and *MALAT1*.

## Discussion

We have for the first time employed next generation sequencing to profile mRNA and lncRNA expression in muscle biopsies from control, IBM and Jo-1 myositis patients. As with all clinical studies based upon biopsies, the differences in mRNA and lncRNA expression is likely to reflect both intrinsic changes within the resident cells, shifts in the proportion of cells resulting from biopsy position and the migration of inflammatory cells. This possibility is supported from our histological studies showing that IBM and Jo-1 biopsies are associated with changes in the muscle structure and infiltration of inflammatory cells (particularly with Jo-1). Given these limitations, we would ideally also perform studies on isolated muscle cells (myocytes) to complement the biopsy analysis. However, the small size of the biopsies precluded the isolation and culture of muscle myocytes. Furthermore, It is also important to highlight that this approach is also problematic since long term myocyte culture might also have altered mRNA/lncRNA expression. Despite the limitations of using biopsies, the histological data indicates that muscle myocytes are the predominant cell type and that this data is likely to provide important new insights into the changes that occur in IBM and Jo1 myositis.

We observed widespread changes in mRNA expression in both IBM and Jo-1 myositis and, as previously reported^24^, pathways analysis indicated that these genes were associated with oxidative phosphorylation and mitochondrial dysfunction. By comparison with the existing lncRNA annotations available in Gencode v23, as well as *ab initio* transcript assembly, we identified 731 known and 325 novel lncRNAs. Of these, the majority were either antisense or located close to protein coding genes (665 lncRNAs) with 391 lincRNAs located between genes. In contrast to previous studies that have indicated a highly significant correlation between expression of lncRNAs and the neighboring mRNAs, this did not appear to be the case in the muscle biopsies^25, 26^.

Comparison with controls showed 55 and 46 lncRNAs were differentially expressed in IBM and Jo-1 myositis respectively, with 16 lncRNAs expressed in both types of myositis. Of the selectively expressed group, none have previously been characterised and future work might examine whether these are important in driving the specific phenotypes associated with IBM or Jo-1 myositis. The 16 lncRNAs that were expressed in both IBM and Jo-1 myositis include a number of characterised lncRNAs such as *H19*, *lncMyoD*, *NEAT1*, *PVT1*, *MEG3* and *MALAT1*, all of which are upregulated. Amongst this group, the imprinted *H19* gene, was previously linked to muscle development and differentiation. A recent report showed that *H19* expression is negatively correlated with bovine fetal muscle mass^27^ and it might be speculated that the increased *H19* in myositis could contribute to the reduced muscle mass and weakness commonly seen in this condition. Increased *H19* expression was detected in cells and tissues derived from patients with other autoimmune diseases, including rheumatoid and osteoarthritis, and is linked to cell de-differentiation and metabolism^28, 29^. As previously mentioned, a number of publications have also shown that *hLncMyoD* regulates muscle proliferation and differentiation^18, 19^. Specifically, Gong *et al*.^19^ found that increased expression of *LncMyoD*, as seen in our myositis samples, was associated with a switch from a proliferative to differentiating muscle phenotype. In the same vein, *PVT1* and *MEG3* were shown to regulate proliferation and cell death in other tissues^30–32^ and future studies might examine whether they have a similar or different role in skeletal muscle.

In conclusion, these studies have shown for the first time that myositis is associated with changes in the profile of lncRNA expression. Based upon the current data, it is not possible to ascertain whether the changes in lncRNA expression are a driver or marker of disease pathogenesis, which will need to be determined through functional studies (including knockdown or over-expression) using cell and/or animal models. The rarity of the IBM and Jo-1 myositis, with estimated combined prevalence of less than 50/1,000,000, means that cell based studies are commonly performed in normal muscle and the results extrapolated to the disease environment. Animal based studies might also be problematic since lncRNA are known to demonstrate poor evolutionary conservation. However, the potential importance of lncRNAs has been highlighted by a recent CRISPR interference based screen of the function of >16000 lncRNAs which identified 499 lncRNA that were required for robust cell growth^33^. We therefore envisage that the information provided in this report provide a basis for future studies of the role of lncRNAs both in normal skeletal muscle and in myositis.

## Patients and Methods

### Patients Biopsies

All muscle biopsies were provided by the Neuropathology Department, North Bristol NHS Trust. Demographic and clinical information was provided with each biopsy sample. Ethical approval was obtained from the National Research Ethics Service Committee East of England and institutional approval was gained from the Royal National Hospital for Rheumatic Diseases Research and Development Committee (REC Reference 12/EE/0068). All experimental procedures were in accordance with the with ethical guidelines. Samples used were linked-anonymised archival biopsy specimens surplus to diagnostic requirements and individual consent was not required for this research.

### Sample Preparation and Next Generation Sequencing

Muscle biopsies were obtained from patients with anti-Jo-1 antibodies (5 patients), IBM (5 patients) and age-matched controls (5 patients). Biopsies were snap-frozen and stored at −80 °C. Following the isolation of RNA fraction (Qiagen RNeasy) samples were examined using an Agilent Bioanalyser and shown to have RIN values 5–7. Following polyA + extraction, samples were subjected to 100 bp paired-end and strand specific sequencing (Illumina TruSeq Stranded Library preparation) on the Illumina HiSeq. 2500 (Wellcome Trust Sequencing Centre, University of Oxford). This produced an average (in millions of reads) of 76, 51 and 74 for the control, Jo-1 and IBM samples, respectively.

### RNA quality control and alignment

Quality control and alignment were performed within the Galaxy Bioinformatics platform. The quality scores across the sequences reads of the raw FASTQ files were assessed using FASTQC v0.9.2 (http://www.bioinformatics.babraham.ac.uk/projects/fastqc). All samples were of high quality giving an average score (mean and median) at each base across reads in each sample of Q > 35. Sequence data was aligned to the human reference genome (hg38) using the following command line options in TopHat2^34^: tophat–library-type fr-firststrand <reference_genome.gtf> −1 <forward_strand.fa> −2 <reverse_strand.fa> -o <output.sam>. Output SAM files were then sorted and converted to BAM files (samtools sort -@ 8 –o output.bam output.sam) and indexed (samtools index –b output.bam) in Samtools^35^. This produced an average of 93% alignment across the control, Jo-1 and IBM samples.

### Identification of novel lncRNAs using RNAseq data

To identify novel lincRNAs and antisense, all potential transcripts were initially assembled from the merged BAM files of the control, Jo-1 and IBM samples by use of Cufflinks2^36^ (Fig. 1a). Bedtools^37^ was employed to identify novel antisense and lincRNAs that did not overlap with genes annotated in GenCode v23^7^. Novel lncRNAs were then identified as those transcripts that contained >1 exon, length >200 bp and which had a low protein coding potential as assessed using the coding potential calculator 0.9-r2.

### mRNA and lncRNA abundance estimation and differential expression analysis

The list of novel antisense and lincRNAs (GTF) was merged with Gencode v23^7^, to produce a master list of mRNA and lncRNAs genes (Fig. 1A). Using this master list (GTF), the profile of gene expression (gene count) in the GTF master list was determined using CuffNorm (V2.2.1.0)^36^ and the data employed for PCA analysis and hierarchical cluster analysis using the Genesis software package (release 1.7.7)^38^. Differential expression of Gencode mRNAs (Supplemental Table 1), Gencode and novel lncRNAs (Supplemental Table 2) was assessed with the geometric option (DESeq) in Cuffdiff v2.2.1.3^39^ using a significance threshold of q < 0.05. The command line options were as follows: cuffdiff–FDR = 0.05–min-alignment-count = 10–library-norm-method = geometric–dispersion-method = pooled –u <reference_genome.gtf> <control_1.bam>, <control_x.bam> <activated_1.bam>, <activated_x.bam> -o <output_file_name> using Cuffdiff (V2.2.1.2). Abundance was expressed as fragments per kilobase exon per million reads mapped (FPKM).

### Determination of evolutionary conservation

A BED file containing all the transcripts for each lncRNAs was extracted from the master GTF files and submitted into the Table Browser Tool on the UCSC genome browser for comparative genomics (https://genome.ucsc.edu/cgi-bin/hgTables).

### Identification of repeat sequences

Repeat sequences were identified and removed from the assembled lncRNA sequences (FASTA) using the default options in Repeatmasker (http://www.repeatmasker.org).

#### qRT-PCR validation of lncRNA differential expression

Expression of mRNAs, lncRNAs and 18S RNA was determined by qRT-PCR using the SYBR® Green PCR mix (Applied Biosystems) and primers obtained from Sigma-Aldrich (Additional File 1). The separate well, 2^−(ΔΔCt)^ method was used to determine relative-quantities of individual mRNAs and lncRNAs relative to18S RNA.

### Data availability

Sequencing data and sample information has been deposited at the GEO repository (GSE102138).

## Electronic supplementary material

Additional File 1

Supplemental Table 1

Supplemental Table 2

Supplemental Table 3
