# Supplementary material for: Transcriptional profiling identifies differential expression of long non-coding RNAs in Jo-1 associated and inclusion body myositis
Source: Sci Rep. 2017 Aug 14;7:8024. doi: 10.1038/s41598-017-08603-9 (PMC5556005; doi:10.1038/s41598-017-08603-9)
Supplement: Supplementary file 1 — Additional File 1 [file 41598_2017_8603_MOESM1_ESM.doc]

**Transcriptional profiling identifies differential expression of long non-coding RNAs in Jo-1 associated and inclusion body myositis**

Philip D. Hamann^1,2,*^, Benoit T. Roux^1,*^, James A. Heward^3,*^, Seth Love^5^, Neil J. McHugh^1,2^, Simon W. Jones^4^ and Mark A. Lindsay^1^

Author Affiliations: ^1^ Department of Pharmacy and Pharmacology, University of Bath, Claverton Down, Bath, BA2 7AY, UK, ^2^ Royal National Hospital for Rheumatic Diseases, Upper Borough Walls, Bath BA1 1RL, UK, ^3^ Barts Cancer Institute, Queen Mary University of London, London, EC1M 6BQ, UK, ^4^ MRC ARK Centre for Musculoskeletal Aging Research, University of Birmingham, Birmingham B15 2TT, UK and ^5^ Dementia Research Group, Institute of Clinical Neurosciences, School of Clinical Sciences, University of Bristol, Bristol BS16 1LE, UK

**Additional File 1**: qRT-PCR probe sequences

| Gene | **Forward** | **Reverse** |
| --- | --- | --- |
| 18s | AAACGGCTACCACATCCAAG | CCTCCAATGGATCCTCGTTA |
| H19 | CATGACATGGTCCGGTGTGA | TAGAAGACAGAGGTCGGGGC |
| IGF2 | CTGGCATCGTTGAGGAGTC | CACGTCCCTCTCGGACTTG |
| CD74 | GACGAGAACGGCAACTTATCTG | GTTGGGGAAGACACACCAGC |
| B2M | GAGGCTATCCAGCGTACTCCA | CGGCAGGCATACTCATCTTTT |
| MALAT1 | ATATGGGGACGTAGGCCGAT | TCTCCAGGACTTGGCAGTCT |
| NEAT1 | TGCTTCATGGACCGTGGTTT | GACTCCATGTCTCCCGGTTC |
| hLncMyoD | TGGAGACAGTTGCTGCATGA | TAGGGGTTCATGGTAGGGGC |
